# The Distribution of Dermatologists in the Philippines

**DOI:** 10.7759/cureus.60402

**Published:** 2024-05-16

**Authors:** Rowena F Genuino, Maria Jasmin J Jamora, La Verne Ivan H Espiritu, Emilio Q Villanueva

**Affiliations:** 1 Department of Anatomy, University of the Philippines Manila College of Medicine, Manila, PHL; 2 Department of Dermatology, Skin and Cancer Foundation, Inc., Pasig, PHL; 3 Department of Pathology, University of the Philippines Manila College of Medicine, Manila, PHL

**Keywords:** health workforce, geographic distribution, health manpower, philippines, dermatologist

## Abstract

Background: Equitable health manpower distribution is essential for the successful implementation of the Universal Health Care (UHC) program by the Philippine Department of Health. Mapping the distribution and profile of dermatologists in the Philippines can improve Filipinos’ access to skin disease treatment.

Methods: A review of the database of dermatologists from the Philippine Dermatological Society (PDS) members’ directory (as of November 2023), as well as the Philippine Health Insurance Corporation (PhilHealth) database (as of July 2023), was conducted. The distribution of PDS-accredited dermatologists was analyzed by geographic location, demographic profile (age and sex), density (per 100,000 people), and the dermatologist-to-general practitioner (GP) ratio. Heatmaps illustrating the distribution of dermatologists in the Philippines and the ratio of PhilHealth-accredited PDS board-certified dermatologists to GPs were created.

Results: Out of 1389 PDS board-certified dermatologists, 1345 resided in the Philippines. The majority were women (1221/1345, 90.78%), with a median age of 47 years (range: 23 to 85). More than half were practicing in the National Capital Region (NCR) (684/1345, 50.86%). The overall dermatologist density was approximately 1 per 100,000 people (1.19); it was highest for the Luzon Island group (1.54) (NCR, 4.80) and lowest for the Mindanao Island group (0.27; the Bangsamoro Autonomous Region of Muslim Mindanao or BARMM, 0.04). Less than one-third (396/1345, 29.44%) of dermatologists were PhilHealth-accredited, with a density of 0.35 dermatologists per 100,000 people. Out of 45218 PhilHealth-accredited physicians, 396 (0.88%) were dermatologists while 11748 (25.98%) were GPs. The overall dermatologist-to-GP ratio among PhilHealth-accredited physicians was 1:30; it was highest in the Luzon Island group (1:20) and lowest in the Mindanao Island group (1:118).

Conclusion: The Philippines lacks dermatologists in regions outside the NCR. The majority are women, and few are PhilHealth-accredited. The dermatologist-to-GP ratio among PhilHealth-accredited physicians is low. Dermatology training programs should encourage more applicants, especially men, and prioritize applicants from underserved regions.

## Introduction

Equitable health manpower distribution is essential to the success of any healthcare system. The Philippines is a lower-middle-income country in Southeast Asia, with a population of 112 million people. It is an archipelago with three island groups (Luzon, Visayas, and Mindanao) composed of more than 7,100 islands. Each island group has a metropolitan area: Metro Manila or National Capital Region (Luzon), Metro Cebu (Visayas), and Metro Davao (Mindanao). The Universal Health Care (UHC) program of the Philippine Department of Health (DOH), approved in February 2019, aims to “protect and promote the right to health of every Filipino” [1]. This can be achieved through a “strong, efficient, well-run health system that meets priority health needs, access to essential medicines and technologies to diagnose and treat medical problems, and a large corps of trained, motivated health workers to provide the services patients need” [2].

Globally, skin and subcutaneous diseases were ranked 21st in the Global Burden of Disease 2019 [3]. The Philippines, together with Malaysia, Thailand, Israel, and Maldives, had more age-standardized disability-adjusted life years (DALYs) caused by skin and subcutaneous diseases than their associated sociodemographic indices (SDIs) in 2017 [4]. In the Philippines, skin diseases accounted for 661.29 DALYs per 100,000 people in 2019 [3].

In general, urban areas have greater dermatologist density, while rural and remote communities may not have a dermatologist. The dermatologist distribution was found to be inequitable in some countries, such as the United States of America (USA), where the density of dermatologists per 100,000 people was 4.11 in metropolitan areas and 1.05 in nonmetropolitan areas in 2013 [5,6]. Similar results were obtained in Brazil [7], Saudi Arabia [8], and Taiwan [9]. In 2020, the highest density of physicians in the Philippines occurred in the National Capital Region (NCR) (100 doctors per 100,000 people), while the lowest density occurred in the Bangsamoro Autonomous Region in Muslim Mindanao (BARMM) during the start of the COVID-19 pandemic (8 doctors per 100,000 people) [10]. Most specialists in the Philippines are concentrated in the NCR, including internists at the Philippine College of Physicians [11].

The Philippine Health Insurance Corporation, or PhilHealth, is a government entity that provides healthcare insurance in the Philippines. Medical case rates for the inpatient management of skin diseases, such as bullous disorders, congenital dermatologic diseases, connective tissue diseases, neoplasms, cutaneous cysts, and skin infections, have increased. PhilHealth also includes procedural case rate packages categorized under the integumentary system, such as incisions and drainage, excisions of benign and malignant lesions, pairing or curettement, shaving of epidermal or dermal lesions, nail procedures, and Mohs micrographic surgery [12]. PhilHealth plays an integral role in implementing UHC in the country, as it aims to provide comprehensive outpatient benefits, including consults and procedures for treating dermatologic concerns, to all Philippine citizens [12]. Hence, by being PhilHealth-accredited, dermatologists can mitigate inpatient out-of-pocket health expenditures.

The Philippine Dermatological Society (PDS) is the only specialty society recognized by the Philippine Medical Association (PMA) and the Philippine College of Physicians (PCP) that specializes in skin, hair, and nails [13]. To be board-certified, one must complete a three-year residency training program, pass the specialty board examination (diplomate), and undergo two years of ethical practice (fellow). It recognizes 15 accredited three-year residency training programs: 13 are in the NCR, one is in Region I (Pangasinan Province) and one is in Region XI (Davao City). The PDS has four local chapters: three in Luzon, namely, Central Luzon (Bataan, Bulacan, Tarlac, Pampanga, Nueva Ecija); northern Luzon (Region I, II, Cordillera Administrative Region or CAR); southern Luzon; and one in the Visayas/Mindanao (southern Philippines). The highest number of skin disease cases seen across all accredited institutions was 93,158 in 2017 based on the PDS-Health Information System (2011-2022) [14].

There are no published studies on the distribution of Filipino dermatologists. There is a need to map the distribution of dermatologists in the country to determine the human resource gaps that need to be filled.

This study aimed to determine the demographic profile (age, sex) of PDS dermatologists and the frequency, percentage, and density (per 100,000) of PDS dermatologists practicing per city/town, province, region, and major island group in the Philippines. In addition, we sought to determine the ratio of PhilHealth-accredited dermatologists to general practitioners (GPs).

## Materials and methods

Study design, setting, and duration

This was a cross-sectional study with information derived from publicly available online data from the PDS website and directory. The PDS members’ directory was accessed through an online PDS portal. PDS members updated their information through the portal at their discretion with no specific set deadline. Data from the PDS members’ directory was retrieved through the PDS secretariat in November 2023. The list of PhilHealth-accredited physicians as of July 31, 2023, was derived from the publicly available online PhilHealth directory, which includes physicians who have successfully registered in PhilHealth as healthcare providers. The study was conducted online and through a review of electronic material publications. The study ran from July 2023 to February 2024.

Eligibility criteria

The inclusion criterion consisted of names listed in the PDS members’ directory as of November 2023. PDS members who were not practicing in the Philippines were excluded. PhilHealth-accredited dermatologists listed in the directory under the categories “Dermatology” and “Dermatology and Cutaneous Medicine” as of July 31, 2023, who were listed in the PDS members’ directory were included. Physicians not listed in the PDS members’ directory, despite being listed under "Dermatology" or "Dermatology and Cutaneous Medicine", were excluded. PhilHealth-accredited GPs listed in the PhilHealth directory under the category “General Practice” were also included.

Sample size and sampling frame

Total enumeration sampling was performed. There were 1389 dermatologists in the PDS members’ directory, 44 of whom were not practicing in the Philippines and were excluded.

There were 45218 physicians in the list of accredited PhilHealth physicians, 11748 of whom were listed under “general practice” (25.98%). A total of 405 accredited physicians were categorized as “dermatology” or “dermatology and cutaneous medicine”, but nine were excluded because they were not members of the PDS; hence, 396 dermatologists were included (0.88%).

Study procedure

The study protocol is available upon request from the authors. The study was exempted from review by the University of the Philippines Manila Research Ethics Board (UPMREB 2023-0660-EX). Permission was obtained from the PDS to access data from the members’ directory. We also collected data from PhilHealth-accredited dermatologists and GPs from the publicly available PhilHealth website. The names of the physicians were anonymized in the data forms by assigning random number codes. We obtained the mid-year population by region as of 2022 from the Philippine Statistics Authority (PSA) [15] to determine the density of dermatologists per region.

Data management and analysis

We extracted data using a pretested data extraction form with the following data items: dermatologist/GP code, age, sex, location of practice (city/town, province, region, major island), and status of practice (practicing or not practicing in the Philippines). For PDS members with two or more locations of practice, we included their primary clinic address. We included all listed members regardless of the completeness of the data. We did not impute missing data on the location of practice or age. We used different denominators in computing the percentages (i.e., age, N1=1211; location of practice, N2=1129). One reviewer collected the data, and a second reviewer checked the accuracy of the data. The data were subsequently entered into a Microsoft Excel spreadsheet (Microsoft® Corp., Redmond, WA, USA).

Data analysis

We determined the frequency, percentage, and density (per 100,000 persons) of dermatologists per province, region, and island group in the Philippines using Stata Statistical Software: Release 17 (2021; StataCorp LLC, College Station, Texas, United States). We created heatmaps of dermatologist distribution using ArcGIS software (https://www.arcgis.com; Environmental Systems Research Institute, Inc., Redlands, USA).

## Results

Out of 1389 board-certified dermatologists listed in the PDS members’ directory as of November 2023, 1345 were practicing in the Philippines. The median age was 47 years (IQR 19). The majority of dermatologists were females (1221/1345, 90.78%) (Table 1). Less than a third of dermatologists were accredited with PhilHealth (396/1345, 29.44%).

**Table 1:** Overall profile of Philippine Dermatological Society board-certified dermatologists in the Philippines as of 2023 (N = 1345)

Profile	Median (IQR); Frequency (%)
Age, years [n=1211]	47 (19)
Sex [n=1345]	-
Male	124 (9.22%)
Female	1221 (90.78%)
PhilHealth Accreditation [n=1345]	-
With	396 (29.44%)
Without	949 (70.56%)

The overall dermatologist density per 100,000 Filipinos is 1.19 (1345 dermatologists for around 112M). More than half of dermatologists who declared a primary clinic address were practicing in the NCR (684/1129, 60.58%) (Table 2) with a density of five dermatologists per 100,000 Filipinos. For the rest of the Philippines, the density of dermatologists was less than one per 100,000 Filipinos.

**Table 2:** Distribution of PDS board-certified dermatologists as of 2023 by primary area of practice PDS: Philippine Dermatological Society; NCR: National Capital Region; CAR: Cordillera Autonomous Region; BARMM: Bangsamoro Autonomous Region in Muslim Mindanao ^a^Source: https://psa.gov.ph/system/files/phcd/2022-12/Cities%2520and%2520Municipalities%2520Population%2520Projections_2015CBPP_Phils.pdf ^b^Total number of dermatologists in the directory with a listed area of practice = 1,129. ^c^Total number of dermatologists in the directory with age data and listed area of practice = 1,216. ^d^Total number of dermatologists in the directory = 1,345.

Region	Population^a^	Overall Density (N2=1129)			Age group (N1=1216)^c^		Sex (N=1345)^d^	
		Frequency	% of total dermatologists^d^	Density (per 100,000 population)	<60y	≥60y	Male	Female
Luzon	64,675,429	994	88.04	1.54	776	216	89	905
NCR	14,262,006	684	60.58	4.80	535	148	58	626
CAR	1,850,151	9	0.80	0.49	7	2	1	8
I	5,390,567	31	2.75	0.58	20	11	1	30
II	3,759,424	19	1.68	0.51	16	3	4	15
III	12,891,548	80	7.09	0.62	63	17	14	66
IV-A	16,938,893	144	12.75	0.85	115	28	11	133
IV-B	3,281,249	8	0.71	0.24	6	2	0	8
V	6,301,591	19	1.68	0.30	14	5	0	19
Visayas	21,252,826	63	5.58	0.30	50	13	6	57
VI	8,092,366	30	2.66	0.37	22	8	1	29
VII	8,233,965	29	2.57	0.35	24	5	5	24
VIII	4,926,495	4	0.35	0.08	4	0	0	4
Mindanao	26,964,526	72	6.38	0.27	57	14	6	66
IX	3,858,548	6	0.53	0.16	6	0	0	6
X	5,180,245	14	1.24	0.27	10	4	0	14
XI	5,531,465	34	3.01	0.61	29	5	4	30
XII	5,107,296	10	0.89	0.20	6	3	1	9
Caraga	2,837,738	6	0.53	0.21	5	1	1	5
BARMM	4,449,234	2	0.18	0.04	1	1	0	2
Overall	112,892,781	1129^b^	-	1.19	956	255	124	1,221

**Figure 1:**
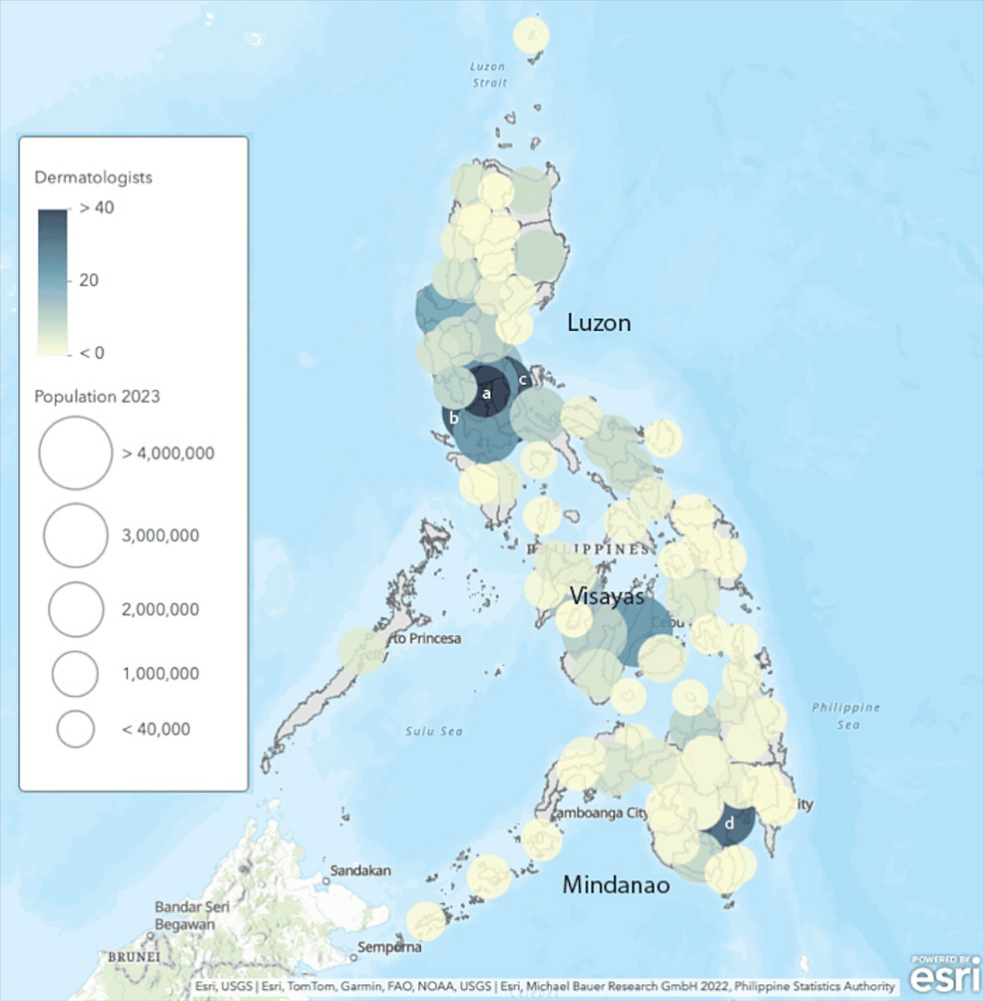
Heatmap showing the distribution of PDS board-certified dermatologists in the Philippines and the midyear population per province as of 2023 ^a^NCR second district ^b^Cavite ^c^Rizal ^d^Davao del Sur Circles represent the population per province, with larger circles representing larger populations. Shading of the circle represents the number of dermatologists within the province, with darker shades representing more dermatologists. Thus, a darker, smaller circle represents a greater density of dermatologists. PDS: Philippine Dermatological Society

As shown by the heatmap, PDS board-certified dermatologists were mostly concentrated in metropolitan areas such as Metro Manila (Luzon Island Group) and surrounding areas, Metro Cebu (Visayas Island Group), and Metro Davao (Mindanao Island Group) (Figure 1).

Among PhilHealth-accredited physicians, the overall density of GPs was 30x higher than that of dermatologists (10.40 versus 0.35 per 100,000 Filipinos), with at least six per 100,000 Filipinos, except for Bangsamoro Autonomous Region of Muslim Mindanao (BARMM) (Table 3). The overall dermatologist-to-GP ratio was 1:30, with the highest (1:6) in NCR and the lowest (1:685) in Region VIII.

**Table 3:** Distribution of PDS dermatologists and general practitioners who are PhilHealth-accredited as of 2023 by primary area of practice ^a^Total number of PDS dermatologists = 1,345. ^b^Total number of PDS dermatologists with PhilHealth Accreditation = 396. PDS: Philippine Dermatological Society; NCR: National Capital Region; CAR: Cordillera Autonomous Region; BARMM: Bangsamoro Autonomous Region in Muslim Mindanao

Region	Population	PhilHealth-accredited dermatologists			PhilHealth-accredited general practitioners		Dermatologist-to-GP ratio
		Frequency	% of total dermatologists^a^	per 100,000 population	Frequency	per 100,000 population	
Luzon	64,675,429	332	33.4	0.51	6,424	9.93	1:20
NCR	14,262,006	231	33.8	1.62	1,300	9.12	1:6
CAR	1,850,151	1	11.1	0.05	386	20.86	1:386
I	5,390,567	17	54.8	0.32	674	12.50	1:40
II	3,759,424	6	31.6	0.16	713	18.97	1:119
III	12,891,548	24	30.0	0.19	1,552	12.04	1:65
IV-A	16,938,893	46	31.9	0.27	1,130	6.67	1:25
IV-B	3,281,249	2	25.0	0.06	272	8.29	1:136
V	6,301,591	5	26.3	0.08	415	6.59	1:83
Visayas	21,252,826	27	42.9	0.13	2,274	10.70	1:85
VI	8,092,366	11	36.7	0.14	505	6.24	1:46
VII	8,233,965	15	51.7	0.18	1,089	13.23	1:73
VIII	4,926,495	1	25.0	0.02	685	13.90	1:685
Mindanao	26,964,526	26	36.1	0.10	3,043	11.29	1:118
IX	3,858,548	4	66.7	0.10	649	16.82	1:163
X	5,180,245	6	42.9	0.12	915	17.66	1:153
XI	5,531,465	13	38.2	0.24	360	6.51	1:28
XII	5,107,296	1	10.0	0.02	409	8.01	1:409
Caraga	2,837,738	1	16.7	0.04	423	14.91	1:423
BARMM	4,449,234	1	50.0	0.02	264	5.93	1:264
Overall	112,892,781	396^b^	29.44	0.35	11,741	10.40	1:30

**Figure 2:**
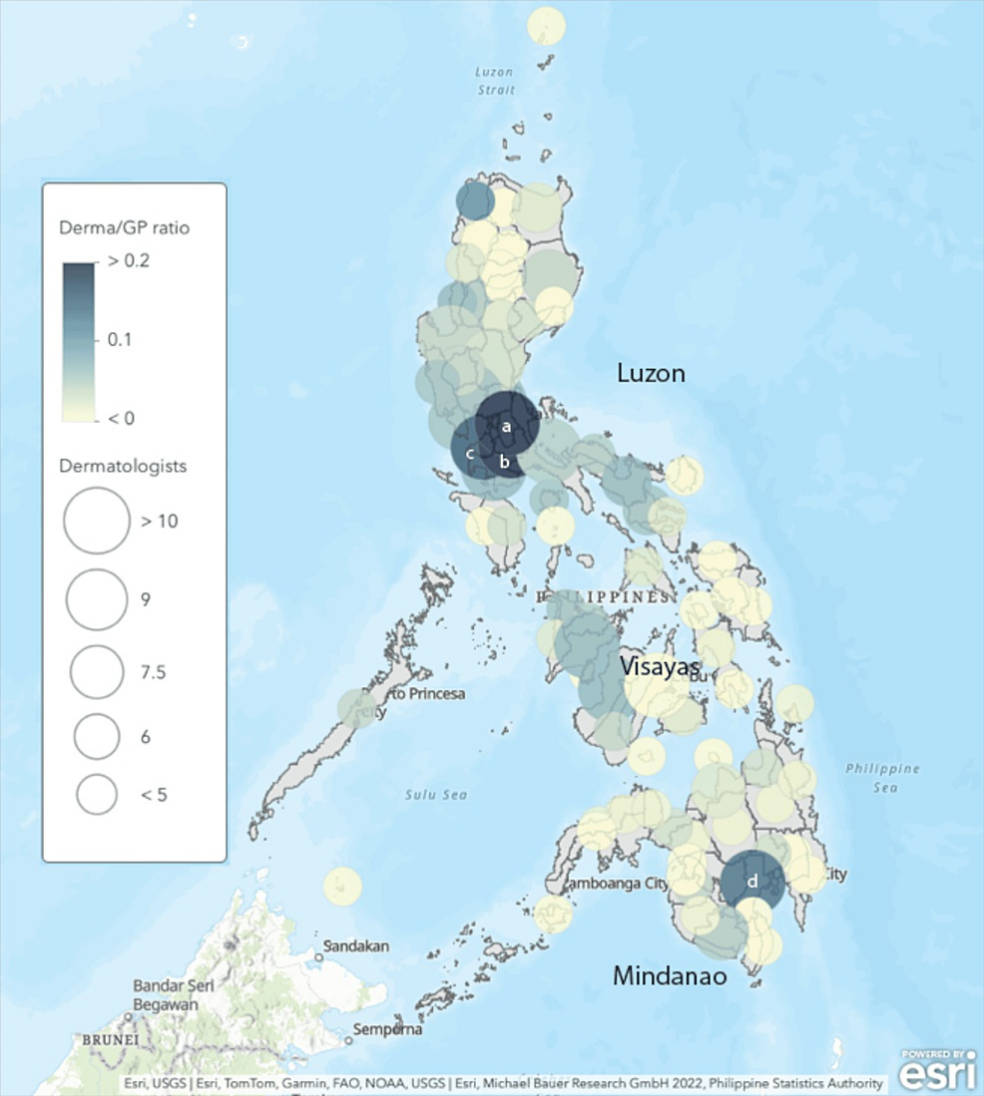
Heatmap showing the ratio of PhilHealth-accredited PDS board-certified dermatologists to general practitioners as of 2023 ^a^Rizal ^b^Laguna ^c^Cavite ^d^Davao del Sur Circles represent the number of PhilHealth-accredited general practitioners per region, with larger circles representing a larger number. Shading of the circle represents the number of PhilHealth-accredited dermatologists within the region, with darker shades representing more dermatologists. Thus, a darker, smaller circle represents a higher dermatologist-to-general practitioner ratio.

The highest ratios of dermatologists to GPs were noted in Rizal, Laguna, Cavite, and Davao del Sur (Figure 2). The lowest ratio was found on the Mindanao Island, followed by the Visayas Island.

Twenty-seven provinces outside the NCR did not have PDS board-certified dermatologists, including 10 provinces in Luzon, seven provinces in Visayas, and 10 provinces in Mindanao (Table 4).

**Table 4:** Provinces outside the National Capital Region (NCR) with and without PDS board-certified dermatologists as of 2023 (n=445) PDS: Philippine Dermatological Society; NCR: National Capital Region; CAR: Cordillera Autonomous Region; BARMM: Bangsamoro Autonomous Region in Muslim Mindanao

Region	Province with dermatologists	Frequency of dermatologists	Provinces without dermatologists
Luzon Island Group (n=310)			
CAR	Benguet	9	Abra, Apayao, Ifugao, Kalinga, Mountain Province
Region I	Ilocos Norte	5	None
	Ilocos Sur	2	None
	La Union	5	None
	Pangasinan	19	None
II	Cagayan	7	Batanes
	Isabela	8	None
	Nueva Vizcaya	3	None
	Quirino	1	None
III	Bataan	9	Aurora
	Bulacan	14	None
	Nueva Ecija	10	None
	Pampanga	34	None
	Tarlac	7	None
	Zambales	6	None
IV-A	Batangas	23	None
	Cavite	35	None
	Laguna	41	None
	Quezon	10	None
	Rizal	35	None
IV-B	Marinduque	1	Occidental Mindoro, Romblon
	Oriental Mindoro	3	None
	Palawan	4	None
V	Albay	7	Catanduanes
	Camarines Norte	2	None
	Camarines Sur	7	None
	Masbate	1	None
	Sorsogon	2	None
Visayas Island Group (n=63)			
VI	Aklan	3	Guimaras
	Antique	1	None
	Capiz	3	None
	Iloilo	13	None
	Negros Occidental	10	None
VII	Bohol	2	Siquijor
	Cebu	22	None
	Negros Oriental	5	None
VIII	Leyte	4	Biliran, Eastern Samar, Northern Samar, Samar, Southern Leyte
Mindanao Island Group (n=72)			
IX	Zamboanga Del Norte	1	Zamboanga Sibugay
	Zamboanga Del Sur	5	None
X	Bukidnon	1	Camiguin
	Lanao Del Norte	4	None
	Misamis Occidental	1	None
	Misamis Oriental	8	None
XI	Davao Del Norte	2	Davao De Oro, Davao Occidental, Davao Oriental
	Davao Del Sur	32	None
XII	Cotabato	1	Sarangani
	South Cotabato	8	None
	Sultan Kudarat	1	None
Caraga	Agusan Del Norte	3	Dinagat Islands
	Agusan Del Sur	1	None
	Surigao Del Norte	1	None
	Surigao Del Sur	1	None
BARMM	Lanao Del Sur	1	Basilan, Sulu, Tawi-Tawi
	Maguindanao	1	None

## Discussion

Among 1389 PDS dermatologists, 1345 or 96.8% were living in the Philippines. The majority were women (90.78%) with a median age of 47 years, with 50.7% practicing in the NCR (4.80 dermatologists per 100,000 population). Less than one-third (n=396, 29.44%) were PhilHealth-accredited. Among PhilHealth-accredited physicians, there were 396 dermatologists and 11,741 GPs, with an overall ratio of one dermatologist to 30 GPs. The dermatologist-to-GP ratio among PhilHealth-accredited physicians was highest in the Luzon Island group (1:20) and lowest in the Mindanao Island group (1:118).

**Figure 3:**
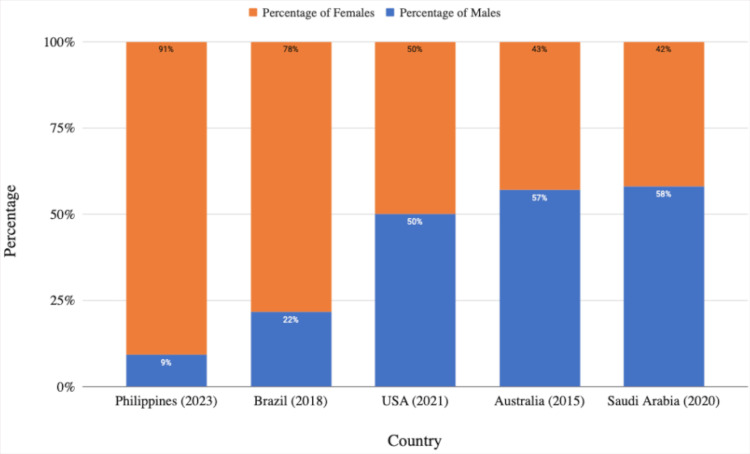
Comparison of the distribution of male and female dermatologists in selected countries

The overwhelming predominance of women among Filipino dermatologists (9 out of 10) was mirrored in Brazil [7]. This contrasts with a nearly equal sex ratio in the US [16] or a slight male predominance in Saudi Arabia [8] and Australia [17] (Figure 3).

Among medical students and trainee residents, dermatology was highly preferred by women in Japan and the USA. In a Japanese study (n=567 medical students), while both sexes ranked internal medicine as their top preference, the preference rates for dermatology, obstetrics and gynecology, and pediatrics were significantly greater for women (19%, ranked 4) than men (<10%, ranked 11) [18]. A US study reported that 61.7% of resident trainees in dermatology were women, ranking sixth in female predominance after obstetrics, gynecology, psychiatry, pediatrics, allergy and immunology, and public health [19]. The characteristics of the dermatology specialty may align well with the preferences and priorities of female physicians in the Philippines. In addition, skin concerns may be more prioritized by women patients, who in turn may prefer dermatologists of the same sex. However, younger Filipino men are also seeking treatment for skin lightening [20] and sexually transmitted infections [21,22]; thus, they may prefer dermatologists who are also men.

The median age of the Filipino dermatologists in this study was 47 years (range, 23 to 85), which is similar to that in Brazil (43, range, 36-54) [7] and Australia (majority 35-64 y/o) [17]. This age group would already represent dermatologists in their first two decades of clinical practice, assuming that they graduated from residency training at the age of 30 years. With the current batch of 198 trainees (personal communication, Ms. Erickha Joy Miguel of PDS, February 5, 2024) representing less than a fifth (n=1345, 14.7%) of the membership, this may ensure that a healthy balance of newly qualified and retiring dermatologists is maintained.

**Figure 4:**
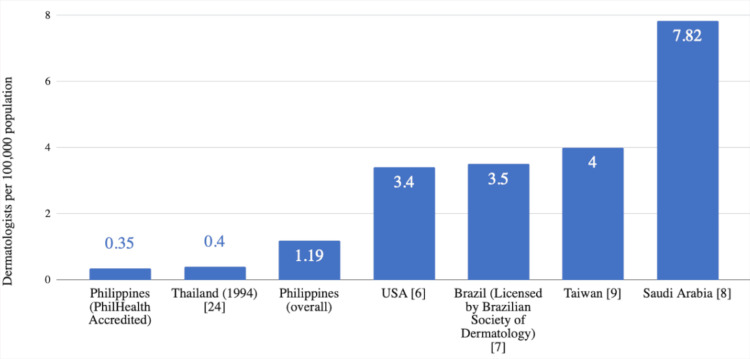
Comparison of dermatologists per 100,000 population in the Philippines versus selected countries

The dermatologist density in the Philippines in this study was 1.19 dermatologists/100,000 people. This falls below the proposed ideal ratio of 3.5 per 100,000 people by Vaidya et al. [23]. Moreover, the dermatologist density among those accredited by PhilHealth was much lower, at 0.35 dermatologists per 100,000 people. Other countries have a higher dermatologist density (per 100,000 people): Taiwan (4.27) [9], the USA (3.4) [6], Brazil (3.5) [7], and Saudi Arabia (7.82) [8]. On the other hand, our overall dermatologist density is greater than that of Thailand (in 1994) (0.4 to 100,000 people) [24] (Figure 4). A lack of PhilHealth-accredited dermatologists may discourage patients from consulting to avoid out-of-pocket expenditures. In addition, the PhilHealth case rate packages for consults and procedures only cover inpatient cases, while dermatologic cases are rarely admitted to the hospital. Thus, there may be a problem with the accessibility and availability of dermatologic care in the Philippines, posing a barrier to the delivery of universal health care.

The clustering of dermatologists in the NCR (4.80 per 100,000) and underrepresentation in the rest of the Philippines (less than 1 per 100,000) is similar to that in Brazil, where less than a 10th (527/5570, 10%) of municipalities in the country have dermatologists [7]. Among the 444 office-based dermatologic clinics in Taiwan, the majority are in urban (n=344, 77.4%) or suburban (20.9%, n=93) areas. Dermatologist-to-population ratios are highest in the cities of Taipei (9.61 per 100,000), Chiayi (6.66 per 100,000), and Taichung (6.23 per 100,000). While 91.3% (n=63) of urban towns had at least one dermatology clinic, 63.9% (n=92) of suburban towns and 96.1% (n=149) of rural towns had none [9]. As of 2018, the highest number of dermatologists (9.2 per 100,000) in Saudi Arabia was in Riyadh, which is one of the two most populated regions [8]. The majority of the Australian dermatology workforce is concentrated in metropolitan areas [18]. Of the 240 qualified dermatologists in Thailand in 1994, 75% practiced in Bangkok, and the other 25% practiced in the provinces [24]. Studies have explored factors that may contribute to physician practices in rural or urban settings. According to Hu et al. (2022), statistically significant factors for respondents from North America include the rural origin of practitioners, locations with better compensation, general familiarity with the area, greater patient need in the area, a similar environment to where he or she grew up, his or her spouse or partner’s job, career advancement, proximity to one’s social network, the quality of schools available for his or her children, and obligations to pre-negotiated programs [25]. Rural practicing physicians were more likely to choose their location if there was better compensation or if they were obliged to pre-negotiate programs. According to a study conducted in the Philippines, as salaries increase, medical interns and graduates place importance on nonwage rural work incentives [26]. Workplace conditions are key factors for doctors to practice in rural settings. Specifically, these include the status of workplace infrastructure, available supplies, relative location of work areas from families, and work supervision. Rural upbringing has also been shown to have a significant bearing on doctors returning to provinces.

There are 27 provinces without any dermatologists: 10 each for Luzon and Mindanao and seven for the Visayas. Notably, all provinces in the NCR have dermatologists, while two regions (CAR and Region XIII/Leyte) have the highest number of provinces (n=5) without dermatologists. Regions with the lowest density of dermatologists, such as BARMM, West Visayas, and Region IX, are also noted to be where the most geographically isolated and disadvantaged area villages and provinces with the highest poverty incidence are located [27]. Similarly, in Taiwan, the majority of suburban (n=92, 63.9%) and rural towns (n=149, 96.1%) did not have a dermatology clinic [9]. All 15 PDS-accredited institutions have conducted teledermatology services, especially during the pandemic, and continue to offer these services through social media such as Facebook as of August 2023 [13]. Among the 15 PDS-accredited institutions, Skin and Cancer Foundation, Inc., has noted that almost half of its graduates have returned to the provinces under its vision of placing dermatologists in underserved areas [13]. Other institutions, such as St. Luke’s Medical Center, Rizal Medical Center, East Avenue Medical Center, and Dr. Jose N. Rodriguez Memorial Hospital and Sanitarium, have also made active efforts to go to referral centers for patients from neighboring provinces [13]. Teledermatology and internet connectivity should be enhanced and institutionalized even after lockdowns have been lifted to ensure access to far-flung areas. In a study by Acoba et al. [28], teledermatology was widely used, particularly during the COVID-19 pandemic. A total of 95.5% (n=113) of the interviewed PDS dermatologists were found to have adequate knowledge and a positive attitude toward this method. In addition to encouraging accredited training institutions to prioritize applicants from these underserved provinces, teledermatology clinics and faster internet bandwidth can be further enhanced in these provinces even beyond the pandemic.

In the Philippines, the PhilHealth accreditation fee for medical specialists was PHP 1,500. The monthly contribution of PHP 2,700, equivalent to PHP 32,400 yearly, is the sample of the contributions of PhilHealth for doctors earning an assumed salary of PHP 60,000 per month as of 2023. The premium rate, however, will increase to 5% by 2024 to 2025. PhilHealth contributions will be required for three years upon the application and renewal of accreditation [12]. The low accreditation rate of Philippine dermatologists (n=1345, 29.22%) heralds a problematic scenario, especially with the upcoming implementation of the UHC Act. This may be because PhilHealth does not cover outpatient care, and dermatologists seldom admit inpatients and have little initiative to seek accreditation.

A study in Ontario [29] revealed that while dermatologic claims made up 3.6% of physician billings under their universal health care system across 17 years, most dermatologic case claims were filed by nondermatologists. Specifically, 24-29% of claims were filed by dermatologists, while 56-62% of claims were filed by nondermatologists. This occurs despite dermatologists seeing 38 times more cases per physician than their nondermatologist counterparts [29]. In contrast, the Netherlands also utilizes a UHC system, and 65.1% (n=326) of skin disease patients are treated by family physicians [29].

The low ratio of one dermatologist to 30 GPs among PhilHealth-accredited physicians is similar to that of Spain in 2012 but lower than that of most European countries, especially Greece (1:1) [30]. This would mean a longer waiting time for Filipino patients who would be referred from the primary care network, which may lead to worse clinical outcomes. To address this disparity, the Philippines needs 12 more dermatologists per GP to match the median ratio (represented by 1:18 for Finland) (Table 5). GPs may also be tapped and trained to provide skin disease treatment in areas without dermatologists.

**Table 5:** Comparison of dermatologist-to-general practitioner ratios in selected countries *Based on data from Trakatelli M, Siskou S, Proby C, et al. [30].

Country	Dermatologist to general practitioner ratio
Greece (2012)*	1:1
Poland (2012)*	1:6
Germany (2012)*	1:8
Italy (2012)*	1:12
Finland (2012)*	1:18
Romania (2012)*	1:19
Netherlands (2012)*	1:20
Malta (2012)*	1:26
Philippines (2023)	1:30
Spain (2012)*	1:30
UK (2012)*	1:57

To address this human resource gap of PhilHealth-accredited dermatologists, the PDS may find ways to encourage PhilHealth accreditation among its members (e.g., discounts on annual membership fees or other incentives) and formalize collaboration with the Philippine DOH for grassroots training and referral networks in the UHC system, as well as with primary care providers’ medical societies.

Limitations of the study include missing data encompassing 216 members without declared locations of practice. Although the PDS Membership Committee has noted that 139 of these are members with inactive statuses or are currently in poor standing, 77 members have not updated their locations of practice in the official database of the PDS; thus, there is a need for a more updated and complete database. Moreover, although the study revealed a lack of PhilHealth-accredited dermatologists in the country, the reasons underlying this lack need to be further explored through interviews and surveys among board-certified dermatologists.

## Conclusions

The Philippine government and the PDS must address geographic disparities in access to dermatologists to plan for equitable health manpower distribution in line with universal healthcare goals. Provinces without dermatologists should be prioritized when accepting new applicants to dermatology training programs. More training institutions are needed, especially in the Visayas and Mindanao island groups. More male dermatologists should be trained. Collaboration between dermatologists and primary care providers, especially in areas with few or no dermatologists, should be facilitated to enable timely referrals. The diagnosis and treatment of common skin diseases through grassroots health worker training must be included to enable quick and appropriate care at the primary health level. Teledermatology and internet bandwidth must be enhanced in remote areas without dermatologists.
